# Comparative differences between T1a/b and T1e/m as substages in T1 urothelial carcinoma of the bladder

**DOI:** 10.1590/S1677-5538.IBJU.2017.0424

**Published:** 2018

**Authors:** Turgay Turan, Özgür Efiloğlu, Bilal Günaydin, Şeyma Özkanli, Emrah Nikerel, Gökhan Atiş, Turhan Çaşkurlu, Asif Yildirim

**Affiliations:** 1 Department of Urology, Istanbul Medeniyet University, Faculty of Medicine, Istanbul, Turkey; 2 Istanbul Medeniyet University, Faculty of Medicine, Department of Pathology, Istanbul, Turkey; 3 Yeditepe Universitesi, Genetics and Bioengineering Istanbul, Turkey

**Keywords:** Carcinoma, Urinary Bladder, Urinary Bladder Neoplasms

## Abstract

**Objective:**

To evaluate the prognostic value of the depth of lamina propria invasion in patients with T1 bladder cancer and to display comparative differences between the T1a/b and T1e/m substaging systems.

**Patients and Methods:**

This study included 106 patients with primary stage T1 urothelial bladder tumours who underwent surgery between January 2009 and December 2014. Pathologic specimens were re-evaluated to confirm the diagnosis of T1 and substaging by the same pathologist using two systems: T1a and T1b, and T1m and T1e. Age, tumour size, multiplicity, associated carcinoma in situ, tumour grade, and T1 substaging system were investigated to detect the relation between disease progression and recurrence.

**Results:**

The recurrence rate was 52% for T1a (n=42) vs. 76% for T1b (n=20) (p=0.028) and 55% for T1m (n=32) vs. 62% for T1e (n=30), respectively (p=0.446). There was no significant difference between the substaging groups for disease progression: T1a (n=12, 15%) vs. T1b (n=7, 27%), and T1m (n=8, 13.8%) vs. T1e (n=11, 23%) (p>0.05). In the multivariate analysis, tumour size >3 cm (p=0.008), multiplicity (p=0.049), and substaging T1b (p=0.043) were independent predictive factors for tumour recurrence. According to the Kaplan-Meier actuarial method, recurrence-free survival was significantly different in patients with pT1a tumours compared with those with pT1b tumours (p=0.033).

**Conclusions:**

Substaging T1 provides a prediction of disease recurrence. Regarding recurrence, T1a/b substaging can provide better knowledge of disease behaviour because it is predicted as more superior than T1 m/e, and it can help in determining the requirement for early cystectomy.

## INTRODUCTION

Bladder cancer is the 7^th^ most common cancer in men; around 79.000 new cases of bladder cancer are estimated to be diagnosed in the United States of America in 2017 (1). Seventy-five percent of these cases are confined to the mucosa or submucosa. The management of stage T1 urothelial bladder tumours can be considered as a therapeutic challenge. Disease progression and death may result from conservative treatment, but radical interventions such as radical cystectomy can be overtreatment for patients with no disease progression. As a result of conservative therapy, disease progression will develop in 20-40% of patients within 5 years (2). Radical cystectomy can be considered as a primary therapy that can be overtreatment for half of the total number of patients (3).

Grade, tumour size, early recurrence, multiplicity, and presence of carcinoma in situ (CIS) are the prognostic factors in T1 urothelial bladder tumours (4). Lamina propria invasion stratification has a high potential risk of disease progression (5). T1 bladder cancer sub-staging has not yet been recommended in clinical guidelines (6). The World Health Organization (WHO) recommended its use according to the recent 2016 classification (7). In addition, there is no consensus as to how to perform sub-staging.

The purpose of the present study was to evaluate the prognostic value of the depth of lamina propria invasion in patients with T1 bladder cancer and to display comparative differences between the T1a/b and T1e/m sub-staging systems.

## PATIENTS AND METHODS

**Figure 1:**
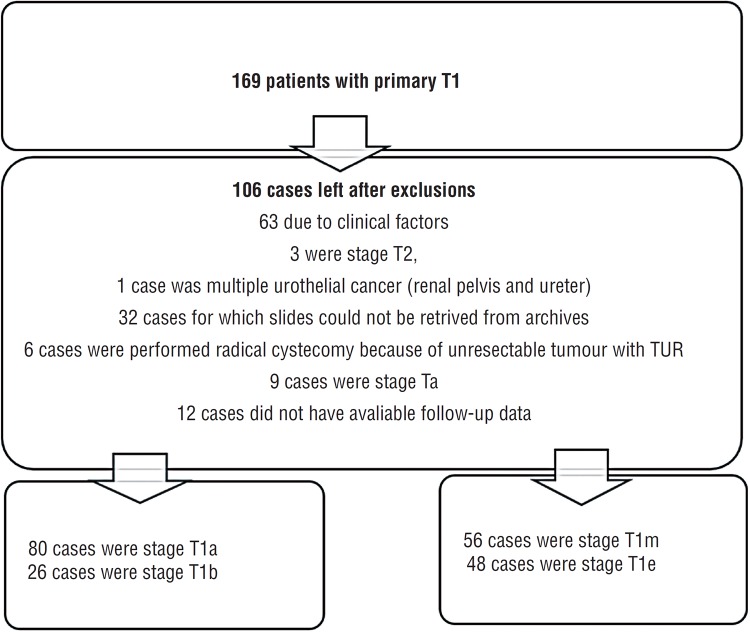
The studied cohort after re-evaluation.

All patients with primary stage T1 urothelial bladder tumours who underwent surgery in our institution between January 2009 and December 2014 were included in the study. To the original study, 169 patients were registered as having primary Tl BC. After re-evaluation and review of the hospital records, a total of 106 patients remained to be included in the analysis (Figure-1). Pathologic specimens were re-evaluated so as to prove the diagnosis of T1 and sub-staging by the same pathologist (SO). The pathologist classified the patients using two systems: T1a (the tumour does not infiltrate the muscularis mucosae-vascular plexus [MM-VP]) and T1b (the tumour infiltrates and/or invades the [MM-VP]), and T1m (micro-invasive- a single focus of lamina propria invasion with a maximum diameter of 0.5mm) and T1e (extensive-invasive, >0.5mm) (8).

All patients underwent macroscopically complete TUR-BT. A second TUR-BT was performed 2-6 weeks after the initial resection in accordance with the recommendations of the current European Association of Urology (EAU) guidelines. Detrusor muscle was present in 96 cases (90.5%) in the first TUR. 10 patients underwent re-TUR and after the evaluation of specimens, it was reported that there was detrusor muscle in all of them. All patients underwent intravesical treatment with bacillus Calmette-Guerin or mitomycin C (BCG or MMC). The patients’ follow-up included cystoscopy and cytology every 3 months in the first 2 years. If no recurrence was found, patients were requested to visit every six months thereafter for 5 years. Disease progression was accepted as the detection of a muscle-invasive BC.

Age, tumour size, multiplicity, associated CIS, second TUR-BT, tumour grade, and T1 sub-staging system were investigated to detect the relation between disease progression and recurrence.

SPSS software version 21.0, was used to perform statistical analyses (IBM, Armonk, NY, USA). Categorical and continuous variables were determined using the t-test, Mann-Whitney U test, and Pearson's Chi-square test. The clinical results, such as disease recurrence and progression, were analysed using univariate statistical analysis according to Kaplan-Meier and multivariate analyses using Cox regression models. A two-sided P<0.05 was considered to indicate statistical significance.

## RESULTS

The mean age of the patients was 67.9±10 years. The mean follow-up time was 54 months. The evaluation of the patients according to staging systems is shown in Table-1. During follow-up, 62 patients experienced recurrence and 19 had disease progression. According to the sub-staging systems, the recurrence rate was 52% for T1a (42 cases) vs. 76% for T1b (20 cases) (p=0.028), and 55% for T1m (32 cases) vs. 62% for T1e (30 cases), respectively (p=0.446) (Table-1). Thus, with regards disease progression, there was no significant difference between sub-staging groups: T1a (n=12, 15%) vs. T1b (n=7, 27%), and T1m (n=8, 13.8%) vs. T1e (n=11, 23%) (p>0.05).

**Table 1:** Characteristics of the analyzed patients according to the two sub-staging systems.

	T1a (n= 80)	T1b (n=26)	p	T1m (n=58)	T1e (n=48)	p
Age	68±10.7	67.7±9.5	0.916	668±10	69.4±10.2	0.203
BMI (kg/m^2^)	26.6±3.4	28.2±43	0.065	26.9±3.2	27±4.2	0.879
Tumor (cm)	3.7±1.9	4.1±1.2	0.372	3.38±1.6	4.38±1.8	0.005^*^
Time to recurrence (mo.)	12.7±13.7	12.4±16.2	0.951	14.7±13.8	10.3±14.6	0.227
Time to progression (months)	13.7±14.7	17.5±20.4	0.603	15.9±16	14±17,2	0.781
Follow-up (months)	44±17.7	43.9±21.5	0.971	45±18.6	42±18.5	0.546
Grade (Low/High)	46/34	3/23	0.001^*^	34/24	15/33	0.005^*^
Percentage of patients who experienced recurrence n (%)	42 (52.5%)	20 (76.9%)	0.028^*^	32 (55.2%)	30 (62.5%)	0.446
Percentage of patients who experienced progression n (%)	12(15%)	7(26.9%)	0.169	8 (13.8%)	11 (22.9%)	0.223
No. of radical cystectomies offered to patients	12 (14.6%)	11 (45.6%)	0.002^*^	6 (10%)	17 (35%)	0.006^*^

During follow-up, 17 (16%) (T0:2, Ta:1, T1:1, T2:10, T3:3) patients underwent radical cystectomy. Radical cystectomy rates for the sub-staging groups were 12.5% vs. 27% for T1a vs. T1b (10 cases vs. 7 cases p=0.082), and 8.6% vs. 25% for T1m vs. T1e (5 cases vs. 12 cases, p=0.022), respectively. Despite our recommendations, six patients refused radical cystectomy surgery due to surgical and/or anaesthetic risks. Nine patients died during follow-up; 7 patients died of tumour progression and 2 died of other causes.

The formulated model of parameters related with recurrence and progression is shown in Table-2. In the univariate analysis, no effect of the sub-staging systems could be determined for disease progression. In multivariate analysis, tumour size >3cm (p=0.008), multiplicity (p=0.049) and sub-staging T1b (p=0.043, OR 0.407 [5-95% CI: 0.173-0.896]) were independent predictive factors for tumour recurrence (Table-2).

**Table 2:** Prognostic factors of tumur recurrence and progression.

	Recurrence univariate analysis	Progression univariate analysis	Recurrence multivariate analysis
	p	p	P
Age	0.652	0.668	0.652
Tobacco Smoking	0.776	0.847	0.676
Second TUR	0.546	0.437	0.588
Low/High Grade	0.894	0.694	0.066
CIS ±	0.142	0.056	0.857
Tumour size >3cm	0.009^*^	0.243	0.008^*^
Multiplicity (single-multiple)	0.0510^*^	0.843	0.049^*^
Sub-staging T1a/b	0.048^*^	0.172	0.043^*^
Sub-staging T1m/e	0.588	0.227	0.588

**Figure 2:**
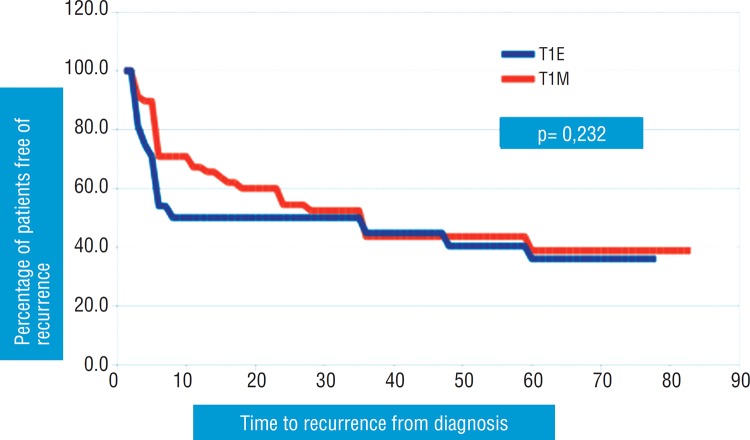
Association of substaging T1m/e and time to recurrence.

According to the Kaplan-Meier actuarial method, recurrence-free survival was significantly different in patients with pT1a tumours compared with those with pT1b tumours (p=0.033). Recurrence-free survival was not significantly different in patients with pT1m tumours compared with those with pT1e tumours (p=0.232). Curves for recurrence-free survival are plotted in Figures 1 and 2.

## DISCUSSION

The management of stage T1 urothelial bladder tumours can be considered a therapeutic challenge. In stage T1 urothelial bladder tumours, progression risk has been reported at different rates. In addition, recurrence following treatment (TURB, second TUR and intravesical treatment) and disease progression cause the necessity for radical cystectomy. In the treatment of high-risk non-muscle invasive bladder cancer, early radical cystectomy is recommended by the EAU guidelines (6). In our study, disease progression was observed in 19 patients and radical cystectomy was recommended to 23 patients. Tumour size >3cm, multiplicity, and T1b were seen as independent risk factors for the prediction of disease recurrence. Sub-staging using T1m/e has no effect on either recurrence or progression. In the multicentre study of Rouprêt et al., it was reported that T1a/b sub-staging was significantly associated with recurrence-free (p=0.03), progression-free (p<0.001) and cancer-specific (p=0.02) survival as a consequence of multivariate analysis (9). In our study, similar results of recurrence-free survival were obtained.

Skoup et al. evaluated the prognostic value of the depth of lamina propria invasion in patients with T1 bladder cancer (10). In that study, during 3.13 years follow-up of 128 patients, the recurrence and progression rates were 61% (n=101) and 16.3% (n=27), respectively. T1 sub-staging and grade also acted as independent predictors of tumour progression in their multivariate analysis (p<0.001). According to the results observed in that study, like in our study, there was no difference in sub-staging using T1a/b and T1m/e (11) on disease behaviour. De-Marko et al. reported that there was no significant diversity between tumour progression and disease-specific survival after 9.5 years of follow-up. However, in classification, the use of T1e/m was recommended, but they did not evaluate recurrence-free survival in their study.

Van Rhijn et al. assessed the T1a/T1b/T1c and T1m/T1e sub-staging systems, which show a higher prognostic value for disease progression and disease-specific survival (8). In their study, sub-staging with T1m/T1e was significant for progression in multivariate analysis. In the present study, however, there was no relation between disease recurrence or progression and T1m/e.

Patriarca et al. analyzed three sub-staging systems for T1 bladder cancers that exhibited different kinds of clinical behavior (T1a/b: 0.5 - 1mm invasion) (12). The authors reported that the 1mm invasion system predicted progression (p<0.04). In the same study, they reported on patients who underwent reTUR; the survival rate without recurrence was satisfactory or more. However, in our study, there appeared no difference because of the performance of nearly standard second TUR.

In the study of Orsola et al., all patients had T1 high grade urothelial carcinoma of the bladder. The authors studied treatment strategies according to sub-staging using depth of lamina propria invasion (5). They showed that sub-staging remained significant for progression on multivariate analysis. In our study, there was no relation between disease progression and the two sub-staging systems. The T1a/b system had a tendency for predicting progression; therefore, a long follow-up period was needed.

In most studies about sub-staging of T1, there have been deficiencies and even failures in reporting. However, we did not encounter such an obstacle. Our study has several limitations. First, the design of the study was retrospective. Secondly, several treatments are available after TURBT (BCG induction therapy, BCG induction, and maintenance therapy, mitomycin C or device-assisted mitomycin C). Our patients did not receive a standardized treatment protocol because there was currently a worldwide shortage of BCG. Thirdly, the limited number of patients and follow-up period may have played a role in the failure of predictive values of sub-staging of T1 for progression. Another limitation to the study is the relative low number of events (62 recurrences) to perform a reliable multivariable analysis with 9 variables. On the other hand, early progression of the disease can be prevented using second TUR, current intravesical induction, and maintenance treatment protocols. In addition, without progression, early cystectomy was performed to several patients. This intervention could bring about a relative decrease in the numbers of disease progression. In our study, tumour size >3cm, multiplicity, and sub-staging T1b for recurrence was very meaningful in univariate and multivariate analyses; however, long-term follow-up might reveal significant diversity for disease progression. Increased numbers of studies and translation of sub-staging of T1 to molecular pathology can lead to more precise prediction of recurrence and progression.

## CONCLUSIONS

Sub-staging T1 in the surveillance of non-muscle-invasive bladder cancer provides the prediction of disease recurrence. Sub-staging of lamina propria invasion depth was designed according to muscularis mucosa retention and these were found as independent factors. Sub-staging should be reported during the pathologic evaluation report because it provides supplementary information for surgeons who undertake the follow-up. Within the assessment of recurrence, T1a/b sub-staging can provide better knowledge about disease behaviour because it is predicted as more superior than T1m/e, and it can also help in determining the requirement for early cystectomy.
